# Multidisciplinary treatment for traumatized refugees in a naturalistic setting: symptom courses and predictors

**DOI:** 10.1080/20008198.2017.1377552

**Published:** 2017-10-10

**Authors:** Nadine Stammel, Christine Knaevelsrud, Katrin Schock, Lena C. S. Walther, Mechthild Wenk-Ansohn, Maria Böttche

**Affiliations:** ^a^ Center Überleben gGmbH (former Center for Torture Victims), Berlin, Germany; ^b^ Department for Clinical-Psychological Intervention, Freie Universität Berlin, Berlin, Germany; ^c^ Refugio Bremen - Psychosocial Care for Refugees e.V., Bremen, Germany; ^d^ Psychologische Hochschule, Berlin, Germany

**Keywords:** Multimodal, refugees, trauma, PTSD, quality of life, therapy, treatment, torture, mental health, transcultural, multimodal, refugiados, Trauma, TEPT, calidad de vida, terapia, tratamiento, tortura, salud mental, transcultural, 多模式, 难民, 创伤, PTSD, 生活质量, 疗法, 治疗, 折磨, 精神健康, 交叉文化, • The current study investigated multidisciplinary treatment for traumatized refugees conducted at a specialized centre for traumatized refugees and torture victims. • The results suggest that the patients benefitted from treatment in terms of improvements of trauma-related symptoms and quality of life. • None of the investigated sociodemographic variables (gender, age, country of origin) significantly predicted the course of the symptoms during treatment, except for somatoform symptoms (younger participants responded better to treatment than older ones).

## Abstract

**Background**: Multidisciplinary treatment approaches are commonly used in specialized psychosocial centres for the treatment of traumatized refugees, but empirical evidence for their efficacy is inconsistent.

**Objective**: In order to obtain more evidence on the development of mental health and well-being of traumatized refugees who receive multidisciplinary treatment, symptom courses of posttraumatic stress disorder (PTSD), anxiety, depression and somatoform symptoms as well as in the subjective quality of life were investigated in the course of a multidisciplinary treatment. In addition, it was analysed if sociodemographic variables were predictors for possible changes in symptomatology and quality of life.

**Method**: *N* = 76 patients of the outpatient clinic of a psychosocial centre for traumatized refugees receiving regular multidisciplinary treatment were surveyed using standardized questionnaires at three measurement points (at the beginning of treatment, and after an average of 7 and 14 months of treatment) in a single-group design.

**Results**: Multilevel analysis showed significant improvements of symptoms of PTSD (*p < *.001), depression (*p < *.001), anxiety (*p < *.001), and somatoform symptoms (*p = .*002) as well as of the subjective quality of life (*p < *.001) over time. Among the tested predictors (gender, age, country of origin), age was a significant predictor for the course of somatoform symptoms (*p *< .05). Younger patients showed greater improvements in symptomatology over time than older ones.

**Conclusions**: The results suggest that the received multidisciplinary treatment had a positive effect on trauma-related symptoms as well as on quality of life of traumatized refugees. There was no indication that sociodemographic characteristics predicted the symptom courses of the patients, except for somatoform symptoms. Younger patients benefitted more from multidisciplinary treatment than older ones.

## Abstract (es)

**Planteamiento:** Los abordajes de tratamiento multidisciplinario se suelen utilizar en centros psicosociales especializados en el tratamiento de refugiados traumatizados, pero la evidencia empírica de su eficacia es inconsistente.

**Objetivo:** Con el fin de obtener más pruebas sobre el desarrollo de la salud mental y el bienestar de los refugiados traumatizados que reciben tratamiento multidisciplinario, en el transcurso de un tratamiento multidisciplinario se investigó la evolución de los síntomas de trastorno de estrés postraumático (TEPT), ansiedad, depresión y somatomorfos, así como de la calidad de vida. Además, se examinó si las variables sociodemográficas predecían posibles cambios en la sintomatología y la calidad de vida.

**Método:** Se realizó una encuesta a N=76 pacientes del ambulatorio de un centro psicosocial para refugiados traumatizados que reciben tratamiento multidisciplinario regular. Para ello, se utilizaron cuestionarios estandarizados en tres puntos de medición (al principio del tratamiento y después de un promedio de 7 y 14 meses de tratamiento, respectivamente) en un diseño de grupo único.

**Resultados:** El análisis multinivel mostró mejorías significativas en los síntomas de TEPT (p <.001), depresión (p <.001), ansiedad (p <.001) y en los síntomas somatoformes (p = .002), así como en la calidad de vida subjetiva (p <.001) a lo largo del tiempo. Entre los predictores probados (sexo, edad, país de origen), la edad fue un predictor significativo para la evolución de síntomas somatomorfos (p <0,05). Los pacientes más jóvenes mostraron más mejoría que los mayores en la sintomatología a lo largo del tiempo.

**Conclusiones:** Los resultados sugieren que el tratamiento multidisciplinario recibido tuvo un efecto positivo sobre los síntomas relacionados con el trauma y sobre la calidad de vida de los refugiados traumatizados. No hubo indicios de que las características sociodemográficas predijeran la evolución de los síntomas de los pacientes, excepto en los síntomas somatomorfos: Los pacientes más jóvenes se beneficiaron más de un tratamiento multidisciplinario que los más mayores.

## Abstract (zh)

**背景：**综合疗法取向在专业的社会心理中心广泛使用于治疗有创伤的难民，但是对其效果的实证证据并不一致。

**目标：**为了获得更多证据了解接受综合疗法的有创伤难民的精神健康发展和幸福感，我们测查了他们在综合疗法中的应激后创伤障碍（PTSD）的症状发展、焦虑、抑郁、躯体形式障碍以及主观生活质量。并同时考察了社会人口统计学变量是否是症状改变和生活质量的预测指标。

**方法：**使用单一组设计，76名在社会心理中心门诊接受常规综合疗法的有创伤的难民，在三个时间点完成了标准化问卷（治疗初期，治疗之后的平均7个月和14个月后）。

**结果：**多水平分析表明PTSD(p <.001)，抑郁(p <.001)，焦虑(p <.001)，躯体形式症状(p =.002)和主观幸福感(p <.001)的显著改进。在测查的预测指标中（性别，年龄，来自国家），年龄对躯体形式症状的病程是一个显著的预测指标(p< .05)。相比于年长者，年轻的患者表现出更强的躯体化症状的改善。

**结论：**结果表明接受综合疗法对有创伤的难民的创伤相关症状和生活质量有积极的改善。除了在躯体形式症状上更年轻患者能够更多地受益于综合治疗外，没有明确的指标说明社会人口统计学变量对患者的病程发展有预测作用。

## 1. Introduction

A growing number of persons worldwide have to flee their homes as a result of war, torture and other systematic human right violations. Refugees are confronted with traumatic experiences in their home countries as well as during their flight, which can lead to severe mental health problems, such as posttraumatic stress disorder (PTSD), depressive, somatoform, anxiety disorders or pain (Bogic, Njoku, & Priebe, 2015; Fazel, Wheeler, & Danesh, 2005; Fuhrer, Eichner, & Stang, 2016; Laban, Gernaat, Komproe, Schreuders, & de Jong, 2004) as well as to physical problems (Forrest, 1999). An average prevalence rate of 30.6% for PTSD and 30.8% for depression was found in populations exposed to mass violence and displacement in a meta-analysis conducted by Steel et al. (2009). Reported torture and cumulative exposure to potentially traumatic events were among the strongest factors associated with both PTSD and depression in those populations. For refugees in Germany, there seem to be comparable prevalence rates for PTSD and depressive disorders (Fuhrer et al., 2016; Gäbel, Ruf, Schauer, Odenwald, & Neuner, 2006), however representative studies are still missing (Bozorgmehr et al., 2016).

Besides mental health problems arising from pre-migration and migration experiences, the living situation of refugees in the host countries is characterized by ongoing stress, which is subsumed as postmigration stress. Postmigration stressors, such as asylum regulation problems, uncertainty about the future, difficult living and social conditions as well as worries about family members in the home countries can interfere with the psychological recovery process and may foster the aggravation and chronification of mental health problems (Laban, Gernaat, Komproe, Van Der Tweel, & de Jong, 2005; Norris, Aroian, & Nickerson, 2011). Despite their overall high level of psychological impairment, refugees have limited access to appropriate medical and psychological care in many western countries, such as Germany, as structural barriers (i.e. communication, language and culture-related problems, lack of information) hinder their access to adequate mental health treatment (Böttche, Stammel, & Knaevelsrud, 2016; Schouler-Ocak, 2015). Treatment is mostly carried out by specialized psychosocial centres for traumatized refugees with limited capacities. In order to tackle the complex problems of their clients (e.g. many traumatized refugees experienced severe and multiple traumatization in their home countries as well as during the flight and suffer from severe and complex forms of PTSD and other trauma-related mental disorders as well as from ongoing postmigration stressors/traumatic events), most psychosocial centres adopt a multidisciplinary approach (often referred to as ‘multimodal approach’, as outlined in Drozdek, 2015; Nickerson, Bryant, Silove, & Steel, 2011) to treat traumatized victims of war and torture. Multidisciplinary treatment involves persons with different professional backgrounds being involved in the treatment process, such as psychiatrists, psychotherapists, social workers or other mental health workers. Multimodal treatment implies that different modules are used by the therapists. Usually multimodal treatment is conducted by a multidisciplinary team. Multidisciplinary and multimodal treatment usually includes culturally sensitive medical, psychotherapeutic and social treatment services, as well as legal assistance for the asylum procedures. Generally, medical doctors, psychotherapists and social workers are included in the treatment process. If necessary, the treatment can be supplemented by other forms of interventions (e.g. body or creative interventions).

Even though the multidisciplinary and multimodal approach is perceived as helpful and feasible by many practitioners, empirical evidence for its efficacy is still missing. So far, there are few and inconsistent results regarding its effect on the symptomatology of traumatized refugees. Mollica and colleagues (1990) describe a multimodal treatment including 52 Southeast Asian refugees consisting of culturally appropriate medication, counselling and social service support carried out by a multidisciplinary team. No significant changes in symptoms of PTSD, depression and anxiety are reported, except for the subgroup of Cambodians showing significant decreases in depression from pre- to post-treatment (see Nickerson et al., 2011; van Wyk & Schweitzer, 2014). An evaluation by Birck (2001) describes a multimodal intervention conducted with mostly Bosnian and Kurdish refugees from Turkey. There were no changes in PTSD, depression and anxiety symptoms from pre- to follow-up assessment; only PTSD-intrusions decreased significantly. However, for most measures the sample size was quite small (ranging from *n* = 7 to *n* = 21). Another multimodal intervention conducted at a specialized treatment centre for refugees in Denmark with refugees from various backgrounds showed no significant symptom changes after an average of nine months treatment (Carlsson, Mortensen, & Kastrup, 2005). After an average of 23 months treatment (Carlsson, Olsen, Kastrup, & Mortensen, 2010), symptoms of PTSD, depression and anxiety decreased significantly from baseline to follow-up, while quality of life did not change significantly. However, the effect sizes of treatment effects were rather low. In this intervention, multimodal treatment included psychotherapy, physiotherapy, social counselling and medical help. In another multimodal intervention conducted by van Wyk and colleagues (2012), the inclusion of exposure based cognitive behavioural therapy (CBT) was explicitly mentioned. They evaluated a naturalistic intervention including 62 Burmese refugees in Australia treated by a multidisciplinary team. Possible treatment elements included psychoeducation, skills-based training, expressive therapy, couples and family therapy, CBT and exposure therapy. The authors report significant changes in symptoms of PTSD, anxiety, depression and somatoform symptoms from pre-assessment to follow-up. To sum up, multimodal approaches show mixed results. As interventions are heterogeneous in terms of applied elements, it is difficult to draw general conclusions. Furthermore, in many studies *t*-tests are applied to compare pre- to post-symptom scores even though the number of treatment sessions, the period of time in treatment or the time of the assessments vary for each patient, making it difficult to generalize the results for all patients.

Only a few studies have investigated the influence of predictors on treatment outcomes for the population of traumatized refugees, revealing mixed results. Most studies examining different possible predictors report no, few or weak factors predicting treatment outcome (Buhmann, Mortensen, Nordentoft, Ryberg, & Ekstrøm, 2015; Haagen, Ter Heide, Mooren, Knipscheer, & Kleber, 2017; Sonne et al., 2016). Sonne and colleagues (2016) found significant but weak correlations between psychosocial resources (such as employment status, lack of social relationships and poor integration) and treatment outcome during a multidisciplinary treatment programme. In terms of sociodemographic variables predicting treatment outcome, the results are mixed. Stenmark, Guzey, Elbert, and Holen (2014) found that female participants responded better to a treatment programme for PTSD than male participants, while other studies did not find gender to predict treatment outcome (Carlsson et al., 2005; Haagen et al., 2017; Stenmark, Catani, Neuner, Elbert, & Holen, 2013). In terms of age being associated with treatment outcome, a study with refugees from Bosnia-Herzegovina suggests that younger refugees might respond better to group treatment and/or medication in terms of PTSD symptom reduction than older ones (Drozdek, 1997), while Carlsson et al. (2005) did not find associations between age and treatment outcome.

Thus, to date, multidisciplinary intervention studies vary considerably in their outcome and they also vary in their methodological quality. Hence, it is a necessary to have more and statistically sound evaluations of multidisciplinary treatment approaches and to analyse factors that can influence the course of treatment. In addition, as practitioners argue that improvements in coping with daily life, such as dealing with postmigration stressors and quality of life, are also a central goal of therapy with traumatized refugees, these concepts should be taken into account in research designs.

To obtain more evidence on the development of mental health and well-being of traumatized refugees who receive multidisciplinary treatment, the current study focused on examining changes in symptoms of PTSD, anxiety, depression and somatoform symptoms as well as in the subjective quality of life in the course of a regular multidisciplinary psychosocial treatment. We thereby aimed to additionally examine patterns of symptom courses over time. A second objective of the study was to examine if sociodemographic variables (gender, age, country of origin) were predictors for possible changes in symptomatology and quality of life. As the literature revealed mixed results concerning the associations of sociodemographic factors and treatment outcome, the research question was analysed in an explorative manner.

## 2. Methods

### 2.1. Participants

Patients (*N* = 76) of the outpatient clinic for adults Center Überleben (former Center for Torture Victims) were surveyed in the context of their regular multidisciplinary psychotherapeutic treatment using a naturalistic study design. The outpatient clinic for adults admits refugees and asylum seekers who were classified as traumatized victims of war and/or torture in need of psychotherapy after an extensive diagnostic phase. All patients received multidisciplinary treatment. The sociodemographic characteristics of the participants are depicted in Table 1.

### 2.2. Setting

The study was conducted at Center Überleben (former center for roture victims) which is a specialized centre for the treatment and rehabilitation of torture victims and traumatized victims of war-related violence. Treatment at the outpatient clinic of the centre follows a multidisciplinary and multimodal approach including culturally sensitive medical, psychiatric, psychotherapeutic and social treatment services that are adapted to the individual needs of patients and, if indicated, supplemented by body and creative therapeutic group modules (Gurris & Wenk-Ansohn, 2013; Wenk-Ansohn, Weber-Nelson, Hoppmann, & Ahrndt, 2014). The psychotherapists at the centre have a medical or psychological background and are trained in cognitive behavioural, psychodynamic or systemic approach and received additional trauma specific trainings. In the current study, *n* = 4 therapists had a cognitive behavioural, *n* = 4 a psychodynamic and *n* = 2 a systemic background. The clinical-social workers have long-term experience in working with traumatized refugees and work in an autonomy-fostering manner.

**Figure 1.**
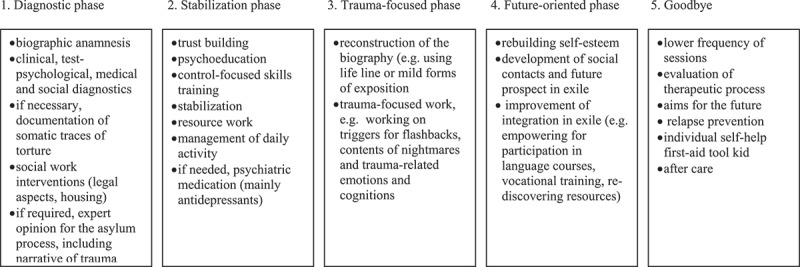
Treatment phases.

After registration and a first screening of the demand on the telephone or in a face-to-face contact, potential patients were assessed in an intake interview by a psychotherapist and a social worker to ensure that only persons who suffer from the consequences of torture or war-related violence were admitted to therapy. Among those, persons who showed pronounced symptoms of trauma-related mental health problems, independent of their residence status, were admitted into treatment in the outpatient centre. Due to limited treatment capacities, only persons with severely and often complex symptomatologies and an urgent need for treatment in an transcultural setting were admitted to treatment. The others were referred to external psychiatrists or to legal support, given that in some cases the impending denial of asylum played a major role for the psychological stress. During the first phase of treatment (the ‘diagnostic phase’, see Figure 1) the first assessment (T0) took place. The diagnostic phase already included some basic interventions (for the elements, see Figure 1). If it became apparent at the end of the diagnostic phase that the clients were not in need or did not have the motivation for a trauma-oriented psychotherapeutic treatment process, they were released from treatment or referred to low-threshold services.

The treatment was conducted based on a phase-model as outlined in Wenk-Ansohn et al. (2014) and Gurris and Wenk-Ansohn (2013). The phases and components of the treatment are depicted in Figure 1. The treatment phases were not applied rigidly, but rather adapted to the individual course of the trauma reactive process, the process of adaptation in exile, the context conditions and the motivation and cultural background of the patient. Treatment sessions were conducted with the help of interpreters who were specially trained to work in psychotherapeutic settings (Wenk-Ansohn & Gurris, 2011). Patients usually had one individual psychotherapy session per week. Sessions with a social worker were conducted additionally dependent on the individual need of the client. If needed, psychotherapeutic and sociotherapeutic crises intervening and stabilizing interventions were applied in a flexible manner, sometimes repeatedly during the course of treatment (e.g. due to severity of trauma and instability of the personality, insecure residential status, postmigration stress or other intercurring retraumatizing/reactualizing events).

### 2.3. Procedures

Patients were surveyed as part of the regularly conducted half-yearly therapy monitoring between June 2007 and May 2013. Data from three measurement points were analysed: T0 at the beginning of therapy, T1 on average 7.2 months (range: 4.4–17.3 months; *SD* = 2.4) after T0, and T2 on average 6.6 months (range: 3.2–15.2 months; *SD *= 2.1) after T1. As some patients were excluded from treatment after T0 we only included participants who had been assessed at least twice, in order to analyse only those clients that were offered psychotherapeutic treatment (see setting). In total, *n* = 91 patients were excluded from the analysis because they completed only the first assessment as they were released before T1 or had incomplete data (e.g. the exact date of the interview was missing).

For all patients included in the analysis, treatment was still ongoing and all were attending multidisciplinary treatment. All measurements were conducted by trained interviewers who were supervised on a regular basis but were independent from the therapists (e.g. trained psychologists or advanced psychology students) supported by a computer-based audiovisual diagnostic tool that allows clients to fill out questionnaires by hearing and reading the questionnaire items in their native language (Multilingual Computer-Assisted Self-Interview, MultiCASI; Knaevelsrud & Müller, 2008). All applied questionnaires had been translated into the native language of the participants and then back-translated and adapted when needed, following a rigorous translation process as recommended for cross-cultural research (Guillemin, Bombardier, & Beaton, 1993). At baseline assessment a structured clinical interview was carried out by the same interviewers with the help of trained interpreters.

### 2.4. Measures

We applied the Mini International Neuropsychiatric Interview (MINI; Sheehan et al., 1998) which is a structured diagnostic interview developed for psychiatric disorders according to the DSM-IV (American Psychiatric Association, 2000) and the ICD-10 (World Health Organization, 2010). For each disorder, between one and four screening questions rule out each diagnosis when answered negatively. The MINI shows satisfactory validity and the reliability scores are reported as acceptable (Sheehan et al., 1998).

PTSD symptoms were assessed with the Posttraumatic Diagnostic Scale (PDS; Foa, 1995; Foa, Cashman, Jaycox, & Perry, 1997). The PDS is based on the diagnostic criteria of PTSD according to the DSM-IV (American Psychiatric Association, 2000). Participants have to rate the frequency of each of the 17 symptom items over the four weeks prior to the interview on a 4-point Likert scale ranging from 0 (not at all or only one time) to 3 (five or more times a week/almost always). The PDS has a high test-retest reliability (Cohen´s kappa = .74), and a high correlation with other instruments for the measurement of posttraumatic symptoms (Foa et al., 1997). We used the sum-score to determine PTSD-symptom severity. As an indication of the validity of the PDS, the clinical cases derived from the PDS were compared to those derived from the MINI at T0. In the current sample, the diagnosis derived from both instruments matched 53 out of 58 (91.4%) participants, while there was a mismatch in only five (8.6%) participants, indicating that the PDS seems to be a valid instrument in our sample to assess PTSD symptoms.

Symptoms of depression and anxiety were measured with the Hopkins Symptom Checklist-25 (HSCL-25; Derogatis, Lipman, Rickels, Uhlenhuth, & Covi, 1974). It consists of a 10-item subscale for anxiety symptoms and a 15-item subscale for depression symptoms. Each item is scored on a 4-point Likert scale ranging from 1 (not at all) to 4 (extremely). The HSCL-25 has been widely used in studies among refugees and diverse cultural groups (Mollica, Wyshak, de Marneffe, Khuon, & Lavelle, 1987; Shrestha et al., 1998). According to Lavik, Hauff, Solberg, and Laake (1999), the HSCL-25 had a high validity in detecting the level of symptoms among traumatized refugees from different cultures. The mean score of both subscales were used as depression and anxiety symptom severity scores, respectively. A cut-off point of >1.75 was applied indicating caseness (Mollica et al., 1987; Winokur, Winokur, Rickels, & Cox, 1984).

Somatoform symptoms were assessed using the 12-item somatization subscale of the Symptom Checklist-90-R (SCL-90-R; Derogatis, Lipman, & Covi, 1973). The checklist captures subjectively perceived impairments by physical and psychological symptoms in the last seven days, which have to be answered on a 5-point Likert scale of distress ranging from 0 (not at all) to 4 (extremely). The sum-score was used to determine somatoform symptom severity.

The European Health Interview Survey 8-Item Index (EUROHIS-QOL-8; Schmidt, Muhlan, & Power, 2006) was used to measure subjective general and global quality of life (QOL). Psychological, physiological, physical, social and environmental facets of quality of life are assessed, each represented by two items ranging from 1 to 5. A cross-cultural study found satisfactory quality criteria and thus a good applicability of the questionnaire in different cultures and languages (Schmidt et al., 2006). The overall QOL score is the sum-score of all eight items, with higher scores indicating better QOL.

### 2.5. Statistical analysis

Missing values in individual items were imputed using individual means to compute scale scores. In order to check if patients who were excluded from data analysis differed from those who were included in the analysis in terms of their symptom severity or quality of life, *t*-tests for independent samples were conducted.

Multilevel analysis (MLA) (Hox, 2010) was applied to assess potential changes in symptoms and quality of life. MLA was chosen to compensate for some variance in the time intervals between the assessments. The number of participants for each assessment and construct as well as the number of participants meeting diagnostic criteria for clinical-caseness for PTSD, depression and anxiety can be found in Table 2.

**Table 1.** Sociodemographic characteristics of participants.

Female gender, *n* (%)	29 (38.2)
Age, *M* (*SD*)	25.4 (10.6)
Self-identified country of origin, *n* (%)	
Iran	25 (32.3)
Chechnya	12 (16.0)
Turkey (Kurdish)	12 (16.0)
Syria	5 (6.7)
Kosovo	4 (5.3)
Afghanistan	4 (5.3)
Iraq	3 (4.0)
Other countries of the Russian Federation	3 (4.0)
Armenia	2 (2.7)
Kenya	2 (2.7)
Angola	1 (1.3)
Chile	1 (1.3)
Lebanon	1 (1.3)
Diagnosis according to MINI at T0, *n* (%)	
Major depressive episode, current	48 (78.7)
Major depressive episode, recurrent	4 (7.0)
Suicidality	22 (36.7)
Manic episode	2 (33.3)
Hypomanic episode	5 (8.3)
Panic disorder	6 (10.0)
Agoraphobia	4 (6.9)
Social phobia	3 (5.0)
Obsessive-compulsive disorder	7 (11.3)
Posttraumatic stress disorder	56 (93.3)
Alcohol dependence (past 12 months)	2 (3.5)
Substance dependence (past 12 months)	3 (5.3)
Psychotic disorders, current	1 (1.7)
Psychotic disorder, lifetime	1 (1.8)
Mood disorder with psychotic features	1 (1.8)
Anorexia nervosa	1 (1.9)
Bulimia nervosa	0 (0.0)
Generalized anxiety disorder	1 (2.5)

**Table 2.** Number of participants, mean scores and clinical cases for PTSD, anxiety, depression, somatoform symptoms and quality of life at the three points of measurement.

Variable	T0	T1	T2
PTSD			
*n*	73	73	45
*M* (*SD*)	36.7 (9.5)	30.0 (10.0)	26.5 (9.6)
*n* (%) ≥ cut-off	72 (98.6%)	69 (94.5%)	42 (93.3%)
Anxiety			
*n*	75	73	43
*M* (*SD*)	2.9 (0.6)	2.6 (0.6)	2.2 (0.6)
*n* (%) > cut-off	71 (94.7%)	67 (91.8%)	35 (81.4%)
Depression			
*n*	76	73	43
*M* (*SD*)	2.8 (0.5)	2.7 (0.7)	2.2 (0.7)
*n* (%) > cut-off	72 (94.7%)	61 (83.6%)	29 (67.4%)
Somatization			
*n*	72	62	39
*M* (*SD*)	26.7 (9.2)	24.3 (8.2)	21.2 (10.0)
Quality of Life			
*n*	72	62	39
*M* (*SD*)	14.6 (5.9)	17.3 (5.3)	21.0 (5.5)

PTSD = Posttraumatic stress disorder. PDS = Posttraumatic Diagnostic Scale. PDS-Cut-off: Rating of 1 or higher by at least one intrusion, three avoidance and two hyperarousal symptoms (according to DSM-IV). HSCL-25 = Hopkins Symptom Checklist-25. HSCL-25- Cut-off: Score of > 1.75. T0 = /T1/T2 = 1st/2nd/3rd point of measurement. Somatoform symptoms were measured with the Symptom Checklist-90-R (SCL-90-R), somatization subscale. Quality of Life was measured with the European Health Interview Survey 8-Item Index (EUROHIS-QOL-8). As there are no cut-offs for the SCL-90-R and the EUROHIS-QOL-8, no numbers of clinical cases can be reported.

The multilevel analysis offers some advantages over other more traditional statistical methods in the evaluation of longitudinal data. Time can be treated as a continuous variable, whereby different time intervals between the points of measurement in individual participants as well as unbalanced data can be utilized in the data analysis (Kwok et al., 2008). For this reason, records of individuals can be included for which no data at every point of measurement is available. The MLA is based on a hierarchical structure of the data, in which the repeated measures in the individual level are formulated as a two-level model (Langer, 2009). Level 1 contains the dependent variables that were measured at the repeated measurements and are nested in Level 2, which represents the individual level (Nezlek, Schröder-Abé, & Schütz, 2006). Finally, an individual growth model is estimated for each person. This consists of the intercept (*γ_00_*) and the slope (*γ_10_*) at level 1. In the present study, the intercept is the value at the beginning of the therapy and the slope represents changes in the dependent variables during therapy.

Individual growth models determine whether, or to what extent, the criterion variable depends on the point of measurement and time-variant or invariant covariates (Langer, 2009). When estimating the coefficients and variances, a distinction is made between fixed and random effects. A fixed effect represents the estimated coefficients for the mean. If a random term for these coefficients is modelled, it demonstrates a random effect (Nezlek et al., 2006). As a result, models with different levels of complexity can be elaborated.

The aim of the calculations in the multilevel analysis is to estimate an equation and subsequent model that best represents the data. The best-known and most frequently used method for parameter estimation is the maximum likelihood function. Furthermore, the likelihood function can be used for the calculation of the deviance, which is used for comparison of nested models (Peugh, 2010). In addition, nested or non-nested models can be compared using the Akaike information criterion (*AIC*) and the Bayesian information criterion (*BIC*) (Kwok et al., 2008). There are no straightforward effect sizes in MLA, but generally accepted indices like the coefficient of determination Pseudo R^2^ can be computed (Raudenbush & Bryk, 2002). Pseudo R^2^ gives the variance explanation by adding one or more predictors compared to a more restrictive model (Peugh, 2010).

The assumptions of the MLA are that the drop-out has to be missing at random (MAR) (Kwok et al., 2008), the deviations of the individual means around the grand-mean (*μ_0j_*) have to be normally distributed and the individual error (*r_ij_*) has to be independent of the random effects between individuals (*μ_0j_*) (Peugh, 2010), that were checked before the analysis.

In the current analysis, longitudinal effects were modelled by the assessment of time coded in months on Level 1. Next, the examination focused upon which growth profile describes best the curve of the dependent variables over time. To this end, random-intercept models with linear growth of each dependent variable were compared with random-intercept models with profiles other than linear (quadratic, polynomial, logarithmic). The models were compared on the basis of the information criteria (*AIC*/*BIC*). The next step was to formulate an unconditioned model for each dependent variable, a random-intercept and a random-slopes model, which were compared in order to determine the model that best fits the data. In the final models, intercepts (*γ_00_*, baseline scores at the beginning of the therapy) and slopes (*γ_10_*, the change in the respective dependent variable in the course of therapy per month), were estimated for every dependent variable.

For those dependent variables with significant variances in the slopes (anxiety, depression and somatoform symptoms), predictors could be inserted into the models at level 2. Thus, the predictors were examined as individual independent variables as well as the interaction between predictors and time. The latter gives information about the potential influence on the growth curves. The demographic variables age, gender and country of origin were checked as possible predictor variables and entered in separated intercept-and-slopes-as-outcome models for every dependent variable.

The variable age was grand-mean centred before inserting it in the models. The 14 countries/regions of origin reported by the participants (see Table 1) were divided into five categories: Iranian (*n* = 25), Arabic (*n* = 13; Afghanistan, Iraq, Lebanon, Syria), Russian Federation (*n* = 15; Chechnya, Dagestan), Turkish/Kurdish (*n* = 12) and rest (*n* = 10; Angola, Armenia, Chile, Kenya, Kosovo). The predictor variable ‘origin’ was dummy coded before inserting it. For this, the unweighted effect coding was chosen because the categories of origin should be equally weighted.

Descriptive data were analysed using the statistic program SPSS 20.0, MLA was computed by the program R (Version3.2.3; R Core Team, 2015) using the package lme4 (Bates, Meaechler, Bolker, & Walker, 2015).

## 3. Results

### 3.1. Comparison of included and excluded patients

No significant differences in the severity of the symptoms and quality of life between patients who were excluded, because they only completed the first assessment, and the included patients were found (PTSD: *t*(156) = −2.51, *p* = .31; anxiety: *t*(164) = −2.06, *p* = .43; depression: *t*(165) = -.67, *p* = .35; somatoform symptoms: *t*(152) = −3.08, *p* = .41; quality of life: *t*(149) = −2.26, *p* = .69).

### 3.2. Multilevel analysis

The assumptions of the MLA were checked and could be confirmed as being fulfilled. It turned out that the symptoms of PTSD could best be described by logarithmic growth, the rest of the variables by linear growth. Thus the following results use logarithmic growth to refer to PTSD and linear growth to refer to the rest of the dependent variables.

The scores of PTSD and subjective quality of life were best represented by random-intercept models (see *χ^2^* Table 3). Thus, the growth curves did not differ significantly between the individuals, only the intercepts varied significantly. The scores of anxiety, depression and somatoform symptoms were best described by random-slopes models (see *χ^2^* Table 3). Thus, for these variables the slopes also varied. Table 3 shows the best fitting model for each construct.

**Table 3.** Results of the multilevel analysis.

	PTSD^a^	Anxiety^2^	Depression^3^	Somatoform symptoms^4^	QOL^5^
*Regression coefficients (fixed effects)*					
Intercept (*γ_00_*)	36.81**	2.94**	2.86**	26.74**	14.68**
95% CI	[26.09,47.52]	[2.17,3.71]	[2.0,3.72]	[12.05,41.42]	[8.4,20.96]
time (*γ_10_*)	−3.64**	−0.04**	−0.04**	−0.36 *	0.42**
95% CI	[−4.75,-2.53]	[−0.13,0.04]	[−0.05,-0.02]	[−1.54,0.81]	[.29, 0.55]
*Variance components (random effects)*					
Residual (*σ^2^_r_*)	67.67	0.38	0.34	4.96	4.65
Intercept (*σ^2^_u0_*)	29.89	0.4	0.44	7.49	3.21
Slope (*σ^2^_u1_*)	-	0.04	0.03	0.6	-
Covariance (*r*)	-	−.21	−.06	−0.41	-
*Variance explanation*					
Pseudo R^2^	.12	.3	.28	.05	.14
*Overall model test*					
*AIC*	1321.51	307.13	292.73	1192.3	1058.06
*BIC*	1334.26	326.32	311.92	1211.1	1070.58
*χ^2^*	107.28**	9.32**	10.36*	10.11*	59.82**

^a^Random-intercept model, logarithmic growth curve, ^2,3 & 4^ random-slopes models, ^5^ random-intercept model 95% CI = 95% confidence interval. Pseudo R^2^: proportional reduction of the total variance by adding the predictor *time_ij_. AIC *= Akaike information criterion, *BIC *= Bayesian information criterion. *χ^2^*: Significance test for comparisons of random-intercept and random-slopes models.

*p < .05, ** *p *< .001.

**Figure 2.**
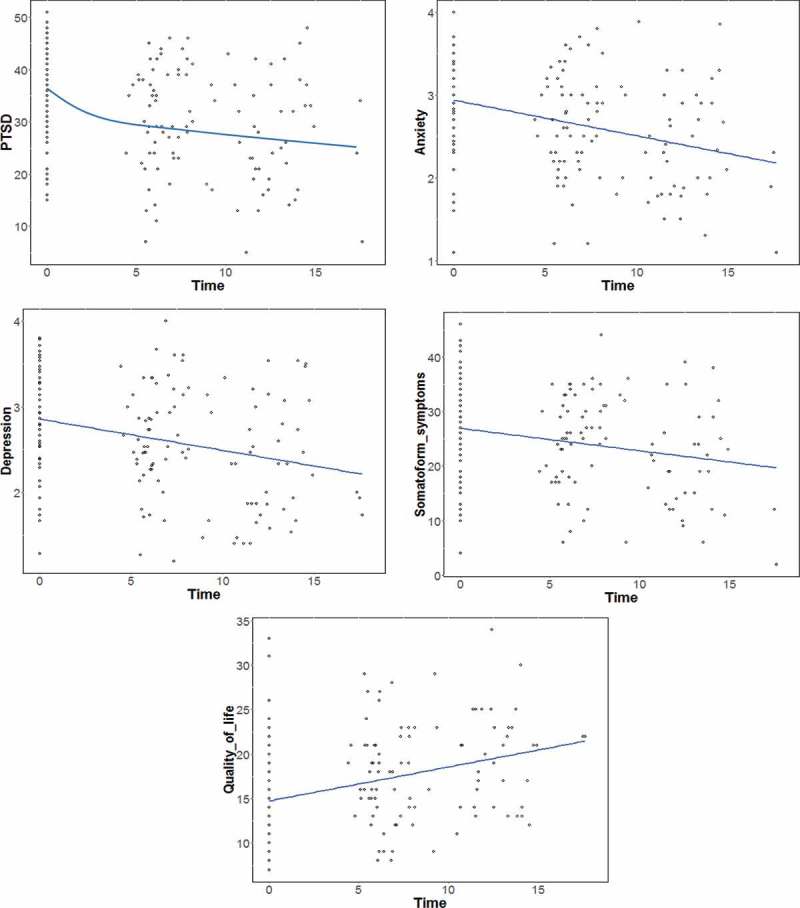
Symptom courses (scatterplots and slopes) for symptoms of PTSD, depression, anxiety, somatoform symptoms and quality of life.

In all final models, time represented a significant predictor for the growth curve of the symptoms (see *y_10_* Table 3), meaning that symptoms of PTSD, depression, anxiety as well as somatoform symptoms and subjective quality of life improved significantly over time. The results of the MLA are shown in Table 3 and are depicted in Figure 2. The Pseudo R^2^ values indicate how much variance is explained by the predictor time. Higher values describe better fits of the models and thus stronger effects of the predictor time.

### 3.3. Demographic variables as predictors

The intercept-and-slopes-as-outcome models showed that the categories of the countries/regions of the participants were not significant predictors of the growth curves for any of the investigated symptom scores (anxiety: *AIC* = 315.54, *BIC *= 366.72, *χ^2^ *= 11.59, *p *= .31; depression: *AIC* = 302.76, *BIC *= 353.94, *χ^2^ *= 9.97, *p *= .44; somatoform symptoms: *AIC* = 1197.9, *BIC *= 1248, *χ^2^ *= 14.4, *p *= .16).

Likewise, gender was not found to be a significant predictor for any of the investigated growth curves (anxiety: *γ_11_ *< 0.01, *SD *= 0.02, *t* = 0.15, *p* = .88; depression: *γ_11_* < −0.01, *SD *= 0.01, *t* = −0.33, *p* = .74; somatoform symptoms: *γ_11_ *= 0.17, *SD *= 0.24, *t* = 0.73, *p* = .47).

**Figure 3.**
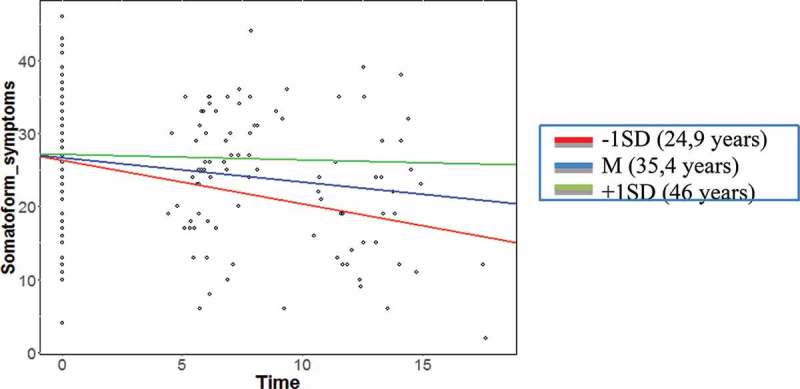
Interaction between age and time for somatoform symptoms.

With respect to age there was no significant interaction with time for the dependent variables anxiety and depression (anxiety: *γ_11_ *= 0.001, *SE* < 0.01, *t* = 1.58, *p* = .12; depression: *γ_12_ *= −0.004, *SD* < 0.00, *t* = 1.55, *p* = .13). However, for the dependent variable somatoform symptoms a significant influence of age on the growth curve could be found (*γ_12_* = 0.02, *SD *= 0.01, *t* = 2.29, *p *< .02) (see Figure 3), indicating that the symptom score of younger participants decreased more strongly over time as compared to older participants. In detail, participants whose age at the first time of measurement (T0) was one standard deviation below the mean age (−1 *SD*, 24.9 years), showed greater improvements than those of average age (35.4 years). The symptoms of participants whose age was one standard deviation above the average (+1 *SD*, 46.0 years) however, showed almost no improvements of their somatoform symptoms. In order to assess the explained variance in the slope between individuals through the level-2 predictor age, Pseudo R^2^ statistics were used. The variance in the slopes explained by adding the predictor age was 22.0%, compared to the random-slopes model.

## 4. Discussion

The aim of the study was to examine changes in trauma-related symptoms as well as subjective quality of life in traumatized refugees in the course of a regular multidisciplinary treatment conducted at the outpatient clinic of a psychosocial centre for traumatized refugees. The results show that the symptom severity of all investigated trauma-related disorders (PTSD, depression, anxiety, somatoform symptoms) decreased, and that the subjective quality of life increased significantly after an average of 14 months of treatment. The results thus indicate that, despite the high symptom load at the beginning of treatment, the patients could benefit from the multidisciplinary treatment. This finding is important as most earlier studies on traumatized refugees receiving multidisciplinary treatment showed no improvements (Nickerson et al., 2011).

To our knowledge only one multidisciplinary treatment study analysed changes in quality of life and found improvements only in the environmental scores from pre- to 9-months assessment (Carlsson et al., 2010). Also in other types of intervention studies, quality of life was rarely investigated and yielded inconsistent results (Buhmann et al., 2015; Ter Heide, Mooren, Kleijn, de Jongh, & Kleber, 2011). Further treatment studies, investigating quality of life or other aspects of coping with everyday life, such as daily functioning, are needed for the population of traumatized refugees and torture survivors.

Despite the significant symptom improvements, the number of clinical cases was still high after one year of treatment (see Table 3). However it has to be considered that predominately severely traumatized persons that suffer chronic and complex disorders were admitted into the therapy programme, treatment was still ongoing for the study participants and many of them lived in stressful life conditions (e.g. living with uncertainty about asylum status). It is well-known that specialized treatment centres often admit treatment-resistant refugees with higher symptom severity (Drozdek, 2015). In fact, the mean scale score of the investigated sample is comparable to those of other intervention studies on traumatized refugees in specialized treatment centres in terms of symptoms of depression, anxiety and PTSD (Carlsson et al., 2010; Neuner et al., 2010; Schock, Bottche, Rosner, Wenk-Ansohn, & Knaevelsrud, 2016). Thus, the results of the current study should be interpreted against this background. Furthermore, high symptom loads after treatment were found in different studies including traumatized refugees (e.g. Buhmann et al., 2015; Carlsson et al., 2010; Neuner et al., 2010), suggesting that even after successful treatment, many patients still meet diagnostic criteria for trauma-related disorders and might still need low-threshold treatment options after therapy. To meet this need and to prevent strong and/or long-lasting symptom relapses (e.g. after distressing news from their home countries or other postmigration stressors), aftercare allowing access to crisis interventions or low frequency therapeutic/psychiatric/social support may be important for patients after completing the initial treatment process.

To our knowledge, the current study is among the first studies that analysed courses of symptoms during multidisciplinary treatment of war and/or torture traumatized refugees. The growth curves showed that PTSD symptoms could be best described by a logarithmic growth, while the rest of the variables were best described by a linear decline. This implies that PTSD symptoms decreased more strongly at the beginning of therapy than at a later stage of therapy, while symptoms of depression, anxiety and somatoform symptoms decreased continuously throughout the course of treatment. At the same time, the subjective quality of life improved continuously throughout treatment. A possible explanation for the early decrease of PTSD symptoms might be that the extensive diagnostic phase which includes the process of working on the trauma narrative might particularly have a positive impact on PTSD symptoms.

A second aim of the study was to analyse if sociodemographic variables (gender, age and origin) were predictors for possible changes in symptomatology and quality of life. Except for somatoform symptoms, the symptom courses were not significantly influenced by the investigated sociodemographic characteristics. Similar to the results of the current study, most studies found no or only weak predictors for symptom change during treatment of traumatized refugees (e.g. Buhmann et al., 2015; Haagen et al., 2017; Sonne et al., 2016). In the current study, the course of somatoform symptoms was predicted by the age of the participants, i.e. younger patients showed a steeper decrease of somatoform symptoms than older ones. It is possible that younger patients responded better to the treatment in terms of the reduction of somatoform symptoms, as symptoms were not yet chronic. This assumption is supported by a study on patients treated at an inpatient unit for persistent somatoform symptoms, showing a positive relationship between a more recent onset of physical symptoms and a better response to treatment (Shorter, Abbey, Gillies, Singh, & Lipowski, 1992). While Drozdek (1997) found younger participants to show stronger reductions in PTSD symptoms, the current study did not find evidence for any of the investigated factors to predict PTSD symptom course. However the studies differ in terms of relating factors (such as the mean age of the participants, the length of received treatment, the origin of the participants), which might have affected treatment outcome. In line with Haagen et al. (2017) and Stenmark et al. (2013), we did not find gender to influence treatment outcome. It can thus be concluded that potential factors influencing the therapy outcome are not yet sufficiently investigated and should be examined more systematically in further studies, including factors such as asylum status, housing, family reunification or personality factors.

### 4.1. Limitations

There are several limitations that need to be considered when interpreting the results. First and most importantly, this study was conducted without a control group. As only persons with severe symptomatologies and an urgent need for treatment were admitted to the centre, the time of waiting for the start of the therapeutic process was kept as short as possible. Because of ethical reasons, it was not possible to implement a randomized design and/or to withhold treatment to a control group for a longer period of time (i.e. for one year as the mean treatment period). Therefore, the shown improvements in symptomatology and quality of life cannot be clearly attributed to the interventions but may also be due to a spontaneous remission of symptoms. The current analysis was conducted in a natural setting with the primary objective to offer treatment for the clients, and the scientific objective was only secondary. The lack of employing control groups and/or randomized controlled designs when investigating multidisciplinary treatment approaches for refugees is a general problem as these approaches are conducted in regular treatment settings where it is often not considered ethical to withhold treatment for scientific purposes (van Wyk & Schweitzer, 2014). Another limitation of the study is that – as in most investigated multidisciplinary approaches – the interventions were not standardized to allow conclusions about which components were eventually responsible for the improvements. When interpreting the results, it should further be considered that the investigated group was highly selective in terms of their level of psychopathology. One has to keep in mind that refugees are only referred to a specialized treatment centre when they are believed to suffer from mental health problems, and only those refugees suffering from high levels of symptom severity and disability are finally admitted to therapy. Drozdek (2015) assumes that this is a general phenomenon when investigating multidisciplinary treatment conducted in specialized treatment centres. Furthermore, treatment was still ongoing for the study participants. Thus, despite the fact that all investigated symptomatologies and quality of life improved over the course of treatment, suggesting that the improvement was quite stable, we do not know if the treatment effects could be maintained, as a post/follow-up assessment could not be carried out for a sufficient number of clients.

## 5. Conclusions

The study evaluates a naturalistic multidisciplinary treatment for traumatized refugees and torture survivors conducted at a specialized treatment centre. After an average of 14 months of treatment, the participants improved on all rating scales on trauma-related mental health problems and subjective quality of life. Despite methodological shortcomings, the study findings are important, as there is only limited and inconsistent evidence for the efficacy of multidisciplinary approaches so far, even though this approach is commonly used in specialized treatment centres for traumatized refugees worldwide. Except for somatoform symptoms and its relation with age, we did not find evidence for sociodemographic factors influencing treatment outcome. Further research is needed on patient characteristics and other factors potentially affecting the success of therapy.

As already proposed by different authors (e.g. Nickerson et al., 2011; van Wyk & Schweitzer, 2014), for future multidisciplinary studies, it would be desirable to implement control groups/randomized controlled trials to ensure that symptom improvements are not due to a natural symptom decline or external factors but caused by the intervention. In addition, more rigorous documentations of treatment components and applied methods as well as external factors (such as changes in residence status) should be implemented in future research designs, which will allow researchers to analyse the impact of the different treatment components and changes in the context factors of the clients on the symptom course.
